# Computed tomography predictors of failure of conservative management in isolated superior mesenteric artery dissection

**DOI:** 10.1007/s11604-026-02023-4

**Published:** 2026-06-13

**Authors:** Sayaka Shirai, Tatsuo Ueda, Fumie Sugihara, Hidemasa Saito, Ryutaro Fujitsuna, Taiga Matsumoto, Misa Iwasaki, Hiromitsu Hayashi, Shin-ichiro Kumita

**Affiliations:** 1 Department of Radiology, Nippon Medical School Hospital, 1-1-5 Sendagi, Bunkyo-ku, Tokyo, 113-8603 Japan; 2 Department of Radiology, Nippon Medical School Tama Nagayama Hospital, 1-7-1 Nagayama, Tama-shi, Tokyo, 206-8512 Japan; 3 Department of Diagnostic Radiology, Kumamoto University Hospital, 1-1-1 Honjo, Chuo-ku, Kumamoto, 860-8556 Japan

**Keywords:** Isolated superior mesenteric artery dissection, Failure of conservative management, Collateral pathways, Riolan’s arch, Endovascular treatment

## Abstract

**Purpose:**

To identify contrast-enhanced computed tomography (CECT) features associated with failure of conservative management in participants with acute-phase isolated superior mesenteric artery dissection (ISMAD).

**Materials and methods:**

This single-center, retrospective, observational study included consecutive patients with acute-phase ISMAD between January 2011 and September 2022. Failure of conservative management was defined as persistent abdominal pain and/or clinical or radiological deterioration despite standardized conservative treatment, prompting close monitoring or consideration of revascularization. The patients were classified into two groups: a failure group (F-G) and a successful group (S-G). The groups were compared with respect to clinical background, Yun classification, true lumen stenosis rate, the cutoff value for the true lumen stenosis rate, and dissection length. Additionally, to evaluate the degree of development of Riolan’s arch (RA), computed tomography images were initially reviewed to confirm visualization, and the maximal vascular diameter was subsequently measured.

**Results:**

Thirty-two consecutive patients with acute-phase ISMAD (median age, 56.7 years; range, 41–81 years; men, n = 26, 81.3%) were included in this study. Nine and 23 patients were classified as F-G and S-G, respectively, according to the study criteria. The median true lumen stenosis rate was significantly higher in the F-G than in the S-G (100.0 vs. 55.0%, *P* < 0.05). Receiver operating characteristic analysis yielded an exploratory cutoff value of 66.8%, with an AUC of 0.855 (95% CI 0.71–0.99); sensitivity, 1.000; specificity, 0.652; positive predictive value, 0.50; and negative predictive value, 1.00. RA was more frequently detected in the F-G than in the S-G (9/9 vs. 12/23, *P* < 0.05).

**Conclusion:**

A high true lumen stenosis rate and the presence of RA on CECT are associated with failure of conservative management in ISMAD and may assist in identifying patients at risk of a clinical course requiring closer monitoring or consideration of revascularization rather than directly predicting intestinal ischemia.

## Introduction

Isolated superior mesenteric artery (SMA) dissection (ISMAD), first described by Bauersfeld in 1947, is a rare disease with a prevalence of 0.01–0.09% in autopsy cases [1]. Recent advances in imaging technologies, such as computed tomography (CT) and magnetic resonance imaging, have increased the number of reported ISMAD cases. ISMAD is a recognized cause of acute abdomen, and most cases can be managed conservatively [2]. However, a subset of patients experiences persistent symptoms or disease progression, necessitating endovascular treatment (EVT) or open surgical intervention. In symptomatic patients, the symptom relief rate ranged from 66.8 to 86.6% after 1 week of conservative therapy [3–6]. Nevertheless, 5.0–44.0% of patients fail conservative treatment or require EVT conversion; moreover, 1.0–3.0% undergo open revascularization due to mesenteric ischemia or necrosis [3, 7, 8].

ISMAD is generally associated with a favorable prognosis, with a reported ISMAD-related mortality rate as low as 0.69% [9]. However, some reports have noted that once acute intestinal necrosis occurs or treatment is delayed, the mortality rate can reach 50–80%, potentially increasing to 80–100% when treatment is delayed beyond 24 h [10]. These findings highlight the importance of prompt and appropriate clinical management strategies.

Although early identification of cases requiring invasive therapeutic intervention is crucial, it is generally difficult to determine which patients are likely to fail conservative management, the pre-stage of intestinal necrosis, using contrast-enhanced CT (CECT) alone. If characteristic imaging findings can be identified and combined with clinical symptoms and dissection morphology, earlier risk stratification and timely therapeutic decision-making can be achieved. Therefore, in this study, we aimed to identify CECT characteristics associated with failure of conservative management in patients with ISMAD.

## Materials and methods

### Study population

This single-center retrospective observational study was approved by the Institutional Review Board of our hospital (Approval Number B-2023-656). All patients provided written informed consent for clinical treatment. Because this was a retrospective analysis, the requirement for informed consent for participation in this study was waived, and patients were notified through public disclosure according to institutional policy.

From January 2011 to September 2022, 46 cases of ISMAD were identified based on morphological findings consistent with ISMAD on CECT. Among these patients, those who demonstrated morphological evidence of ISMAD on CECT and presented with abdominal pain attributable to the dissection were classified as acute-phase ISMAD. Patients whose abdominal pain was clearly attributable to other identifiable causes, those suspected to be in the chronic phase, or those with incomplete clinical or imaging data were excluded. Failure of conservative management was defined as persistent abdominal pain lasting more than 7 days and/or clinical or radiological deterioration despite standardized conservative treatment, including bowel rest, blood pressure control, analgesia, and antithrombotic therapy when indicated. Based on this definition, patients were classified into a failure of conservative management group (F-G) and a successful conservative management group (S-G).

### Image acquisition

All CT examinations were performed on multidetector CT scanners: 64-row (Optima™ CT600 and LightSpeed™; GE Healthcare, Chicago, IL, USA), 128-row (Somatom™ Definition AS+ ; Siemens Healthineers, Erlangen), 256-row (Revolution™; GE Healthcare, Chicago, IL, USA), and 320-row (Aquilion™ ONE; Canon Medical Systems, Otawara, Japan).

All patients underwent multiphase contrast-enhanced abdominal CT, including non-contrast, arterial, and portal venous phases. First, a non-contrast scan was performed. For the contrast study, a non-ionic iodinated contrast medium (approximately 1.5 mL/kg) was intravenously injected at a rate of 3–4 mL/s. The arterial phase was acquired approximately 25–35 s after the start of contrast injection, and the portal venous phase was obtained at 60–70 s. These timings were either fixed or triggered by bolus tracking in the abdominal aorta or the superior mesenteric artery to ensure optimal vascular enhancement. The detailed imaging parameters were as follows: tube voltage, 120 kV; tube current, 150–500 mA depending on the body mass index; gantry rotation speed, 0.4–0.5 s/rotation depending on the CT scanner; collimation, 64 × 0.5 mm; and helical pitch, 0.984. Non-contrast CT images of the SMA were obtained at a slice thickness of 5 mm, and CECT images (arterial and portal venous phases) were acquired at a slice thickness of 0.625–5.0 mm. All images were reviewed independently by two experienced radiologists; each measurement was performed twice, and the average value of the two measurements was used for analysis.

### EVT procedure

Under local anesthesia, either the right or the left common femoral artery was punctured with an 18-G needle (Catheter Introducer; Medikit, Tokyo, Japan), and a 4-Fr sheath (Supersheath; Medikit) was inserted. After performing SMA angiography, the sheath was exchanged for a 6-Fr guiding sheath (Destination™, Terumo Corporation, Tokyo, Japan). Heparin (3000–5000 units) was administered intravenously based on the patient’s body size. A 0.035-in guidewire (Radifocus Guide Wire M; Terumo, Tokyo, Japan) was advanced beyond the dissected segment to the distal SMA, the guiding sheath was then advanced to the distal end of the dissection, and a bare-metal stent (LifeStent™, Bard Peripheral Vascular, Tempe, AZ, USA, or SMART™ Control, Cordis Corporation, Miami Lakes, FL, USA) was deployed. The stent landing zone was selected to fully cover the dissected segment, as confirmed by angiography, and the stent diameter was chosen based on the total SMA external diameter at the dissected site. In principle, even when the dissection extended into the branch vessels, the stent was deployed across the branches and positioned on the main SMA trunk. Postdilatation with a balloon catheter was not performed after stent deployment.

### Efficacy assessment and definition

As mentioned earlier, failure of conservative management was defined as persistent abdominal pain (> 7 days) and/or clinical and/or radiological deterioration requiring escalation to invasive management, and patients were accordingly classified into the F-G and S-G. Radiological deterioration was defined as progression of the extent of dissection, new involvement of branch vessels, newly developed hypoenhancement of the bowel wall, or bowel wall edema on follow-up CECT. Importantly, the imaging predictors evaluated in this study (true lumen stenosis rate and development of Riolan’s arch [RA]) were not used for outcome adjudication.

The following data were collected and compared between the F-G and S-G: demographic factors (age, sex, and presence or absence of hypertension, dyslipidemia, diabetes mellitus, and smoking history), hospitalization duration, follow-up duration, treatment method, dissection morphology according to Yun’s classification [11], true lumen stenosis rate, dissection length, distance from the SMA origin to the dissection entry point, presence or absence of dissection extension into peripheral branches, and presence or absence of increased perivascular adipose tissue density around the dissection site. Yun’s classification was selected because it is widely used in clinical practice and provides standardized morphological stratification of ISMAD, thereby facilitating comparison with prior studies. The true lumen stenosis rate (%) was measured at the maximal narrowing site and calculated as (1–true lumen diameter/total SMA diameter) × 100.

To ensure consistency and reproducibility, measurements were performed on multiplanar reformatted images perpendicular to the centerline of the SMA. Diameter-based measurements were used consistently, acknowledging their limitations in cases of eccentric compression. The dissection length and distance from the SMA origin to the dissection entry point were measured on sagittal CECT images.

The presence or absence of a developed RA was assessed on arterial-phase CECT images with a slice thickness of 0.625–5.0 mm, and its maximum diameter was measured. The presence of RA was defined as a continuous arterial connection between SMA and the inferior mesenteric artery (IMA) that could be traced along its entire course on arterial-phase CECT images, including multiplanar reconstructions when necessary. RA presence and maximum diameter were separately evaluated. Interobserver agreement for RA diameter measurements was assessed using the intraclass correlation coefficient (ICC). Interobserver agreement for RA presence was assessed using Cohen’s kappa coefficient. The presence and maximum diameter of RA were independently evaluated by two board-certified radiologists who reviewed all images while having access to baseline clinical information but not to treatment allocation or outcomes. Discrepancies were resolved by consensus.

Moreover, the technical and clinical success rates, complications, and mortality rates of EVT were evaluated. Technical success was determined as the restart of antegrade flow in the true lumen on the final angiography following stent deployment. Clinical success was identified as abdominal pain resolution after treatment. According to the Society of Interventional Radiology classification, complications were defined as major complications requiring therapy [12]. Mortality was defined as death from any cause related to ISMAD or treatment during hospitalization.

### Statistical analyses

Statistical analyses were performed using the EZR software. Continuous variables are expressed as medians and interquartile ranges (IQRs), and categorical variables are expressed as numbers and percentages. Continuous variables between the F-G and S-G were compared using the Mann–Whitney *U* test, and categorical variables were compared using the chi-square test or Fisher’s exact test, as appropriate. For the true lumen stenosis rate, a receiver operating characteristic (ROC) curve was generated to determine the optimal cutoff value, along with the 95% confidence intervals (CIs) for the area under the curve (AUC), sensitivity, specificity, the positive predictive value (PPV), and negative predictive value (NPV). Statistical significance was set at *P* < 0.05.

## Results

### Patient characteristics

During the study period, from January 2011 to September 2022, 46 patients were diagnosed with ISMAD based on CECT at our institution. The clinical characteristics of the F-G and S-G are summarized in Table 1.

**Table 1:** Clinical characteristics of patients in the failure and successful groups

	Failure group	Successful group	*P*
	(n = 9)	(n = 23)	
Age (years), median (IQR)	58 (51–72)	55 (48–60)	0.57
Sex (M / F)	6/3	20/3	0.31
Hypertension, No. (%)	3 (33.3)	12 (52.2)	0.44
Dyslipidemia, No. (%)	4 (44.4)	8 (34.8)	0.70
Diabetes, No. (%)	2 (22.2)	3 (13)	0.60
Smoking, No. (%)	7 (77.8)	14 (60.9)	0.44
Hospitalization days (days), median (IQR)	21 (12–25)	8 (0–36)	< 0.05
Follow-up duration (days), median (IQR)	502 (134–1066)	641 (215–1080)	0.80
Conservative treatment, No. (%)	2 (22.2)	23 (100)	< 0.05
Endovascular treatment, No. (%)	7 (77.8)	0 (0)	< 0.05
Antithrombotic therapy, No. (%)	7 (77.8)	12 (52.2)	0.25

IQR, Interquartile range; Failure group, group with failed conservative management; Successful group, group with successful conservative management

**Fig. 1:**
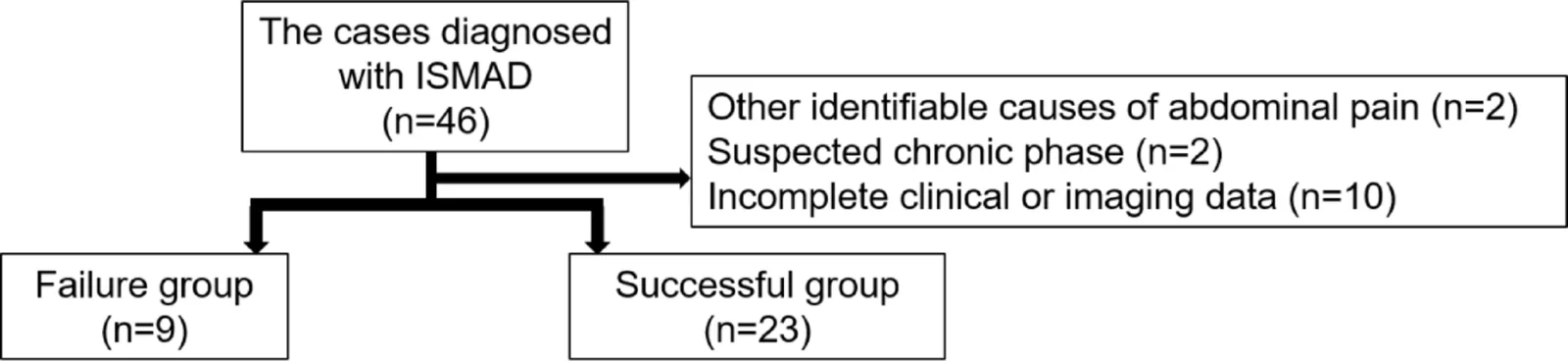
Participant selection flowchart. Among 46 patients diagnosed with ISMAD on contrast-enhanced CT between January 2011 and September 2022, 14 were excluded for the following reasons: suspected chronic phase (n = 2), suspected other identifiable causes (n = 2), and incomplete clinical or imaging data (n = 10). The remaining 32 patients with acute-phase ISMAD were included and classified into a failure of conservative management group (F-G) and a successful conservative management group (S-G). ISMAD, isolated superior mesenteric artery dissection; CT, computed tomography

In total, 14 patients were excluded (two patients with abdominal pain caused by other diseases, two considered chronic, and 10 with incomplete clinical or imaging data). The remaining 32 patients with acute-phase ISMAD were included in this analysis; among them, nine and 23 were classified into the F-G and S-G, respectively, according to the study criteria (Fig. 1).

In the F-G, the median age was 58 years (range, 51–72 years), and six patients (66.7%) were male. In the S-G, the median age was 55 years (range, 48–60 years), and 20 patients (87.0%) were male.

No statistically significant differences were observed between the two groups regarding the prevalence of hypertension, dyslipidemia, diabetes mellitus, smoking history, or follow-up duration. Hospitalization duration was significantly longer in the F-G than in the S-G. All patients in the S-G were treated conservatively. No significant differences were observed regarding antithrombotic therapy.

### Clinical outcome

**Fig. 2:**
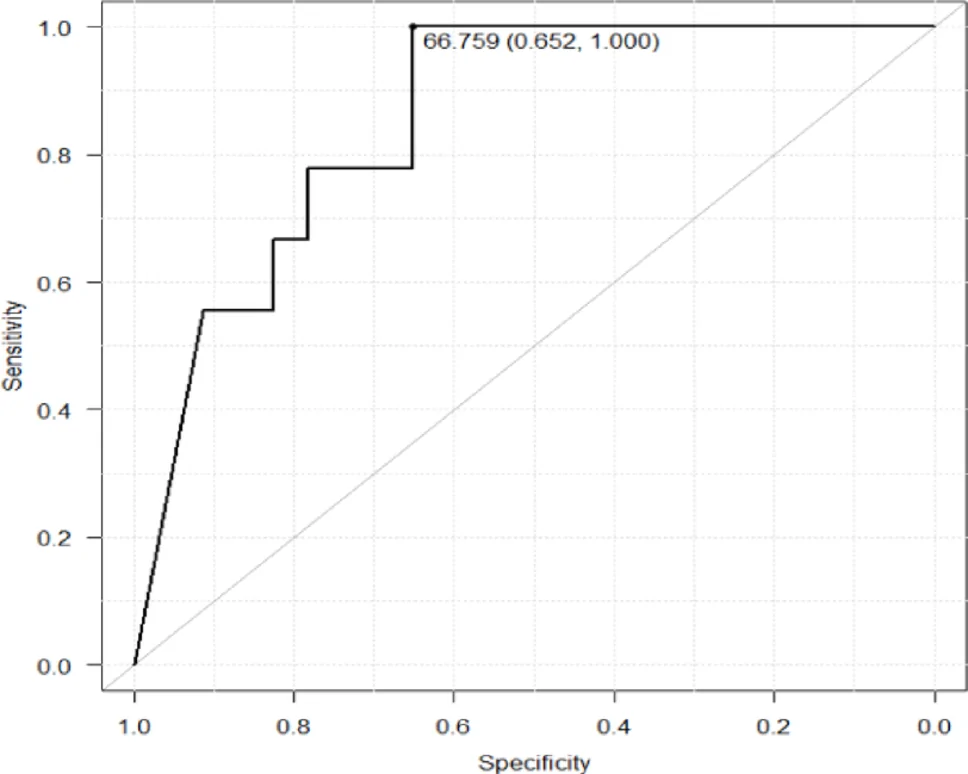
ROC curve for the true lumen stenosis rate. ROC curve for the true lumen stenosis rate as a predictor of failure of conservative management in ISMAD. The area under the curve is 0.855. An exploratory ROC-derived cutoff value of 66.8% is shown. ROC, receiver operating characteristic; ISMAD, isolated superior mesenteric artery dissection

The CECT findings of the F-G and S-G are summarized in Table 2. Morphological evaluation according to Yun’s classification revealed that type III dissections, characterized by severe narrowing or obstruction of the true lumen, were significantly more frequent in the F-G group than in the S-G group, whereas type IIb dissections were more common in the S-G group than in the F-G group. The median true lumen stenosis rate was significantly higher in the F-G than in the S-G (100.0 vs. 55.5%, *P* < 0.05). ROC curve analysis for predicting failure of conservative management identified an exploratory ROC-derived cutoff value of 66.8% for the true lumen stenosis rate, with an AUC, sensitivity, specificity, PPV, and NPV of 0.855 (95% CI 0.71–0.99), 1.000, 0.652, 0.50, and 1.00, respectively (Fig. 2). No significant differences were found between the two groups regarding dissection length, distance from the SMA origin to the dissection entry point, or presence of increased perivascular adipose tissue density. Nonetheless, dissection extension into the peripheral branch vessels was observed significantly more often in the F-G than in the S-G (*P* < 0.05).

**Table 2:** Enhanced computed tomography findings in the failure and the successful groups

		Failure group	Successful group	*P*
		(n = 9)	(n = 23)	
Yun’s classification, No. (%)	Type Ⅰ	1 (11.1)	1 (4.3)	0.52
	Type Ⅱa	0 (0)	1 (4.3)	0.58
	Type Ⅱb	3 (33.3)	19 (82.6)	< 0.05
	Type Ⅲ	5 (55.6)	2 (8.7)	< 0.05
The true lumen stenosis rate (%), medidan (IQR)		100 (74.8–100)	55.5 (45.0–72.9)	< 0.05
The dissection length (mm), medidan (IQR)		29.4 (9.5–55.4)	60.5 (40–73.8)	0.18
The distance from the origin of the SMA to the dissection entry point (mm), medidan (IQR)		25.0 (6.4–27.0)	10.6 (5.7–19.3)	0.11
The presence or absence of dissection extension into peripheral branches, No (%)		9 (100)	12 (52.2)	< 0.05
The presence or absence of increased perivascular adipose tissue density around ISMAD, No (%)		5 (55.6)	15 (65.2)	0.70

IQR, Interquartile range; ISMAD, Isolated superior mesenteric artery dissection; Failure group, group with failed conservative management; Successful group, group with successful conservative management

Table 3 presents a comparison of RA presence and RA diameter between the F-G and S-G, analyzed as separate imaging parameters. With respect to collateral circulation, a developed RA was identified in all nine patients (100%) in the F-G, compared with 12 of 23 patients (52.2%) in the S-G (*P* < 0.05).

**Table 3:** Comparison of the presence and degree of development of Riolan’s arch between the failure and successful groups

	Failure group	Successful group	*P*
	(n = 9)	(n = 23)	
RA development, No. (%)	9 (100)	12 (52.2)	< 0.05
The maximum diameter of RA (mm), median (IQR)	2.6 (2.3–3.0)	2.3 (1.7–2.8)	0.18

IQR, Interquartile range; SD, Standard deviation; RA, Riolan’s arch; Failure group, group with failed conservative management; Successful group, group with successful conservative management

The maximum RA diameter showed no significant difference between groups, measuring 2.6 mm (IQR, 2.3–3.0 mm) and 2.3 mm (IQR, 1.7–2.8 mm) in the F-G and S-G, respectively. The interobserver agreement for RA presence was substantial, with a Cohen’s kappa value of 0.80. The agreement rate was 90.6% (29/32 cases), with three discrepant cases before consensus. The interobserver agreement for maximum RA diameter measurements was good, with ICC of 0.88 (95% CI 0.75–0.95). Within the F-G, seven patients underwent EVT. The technical and clinical success rates were 100%, with no procedure-related complications. The mortality rate in this study was 0% (Table 4).

**Table 4:** Outcomes in the endovascular treatment group

	EVT group (n = 7)
Technical succcess rate (%), no. (%)	7 (100)
Clinical success rate, no. (%)	7 (100)
Complication rate, no. (%)	0 (0)
Mortality, no. (%)	0 (0)

EVT, Endovascular treatment

## Discussion

Here, we identified two novel and clinically relevant CECT markers—true lumen stenosis rate and RA development—which were associated with failure of conservative management in patients with ISMAD. Rather than predicting histologically confirmed intestinal ischemia, this study focused on identifying imaging markers of a clinically unstable state that necessitates close monitoring or consideration of revascularization. Our results align with those of previous studies that reported a favorable prognosis for most ISMAD cases with conservative therapy as the first-line approach [11]. In a meta-analysis by Karaolanis et al. [13], conservative management was applied in 72% cases, although 16.7% eventually required angioplasty. Moreover, earlier studies have shown that 5–44% of initially conservatively managed patients required EVT conversion, and 1–3% underwent open revascularization for mesenteric ischemia or necrosis [3, 7, 8]. In our cohort, seven of nine patients in the F-G (77.8%) underwent EVT. Of the remaining two patients, one was suspected of having intestinal ischemia based on imaging findings and underwent exploratory laparotomy; however, no definitive ischemic changes were identified. It may be possible that transient ischemia occurred initially; however, it did not progress further due to the development of collateral pathways. This case underscores the importance of integrating imaging findings with other clinical and laboratory assessments when evaluating acute-phase ISMAD, as imaging alone may be insufficient for accurate diagnosis. Because the study endpoint reflects escalation to invasive management, which may be influenced by clinical decision-making and imaging severity, the findings should be interpreted as associations with the clinical course rather than direct predictors of intestinal ischemia. Another patient had a psychiatric comorbidity, and their self-reported pain was inconsistent. Psychiatric comorbidities may have influenced symptom perception, as previous reports indicate that psychiatric conditions can alter pain experience [14].

**Fig. 3:**
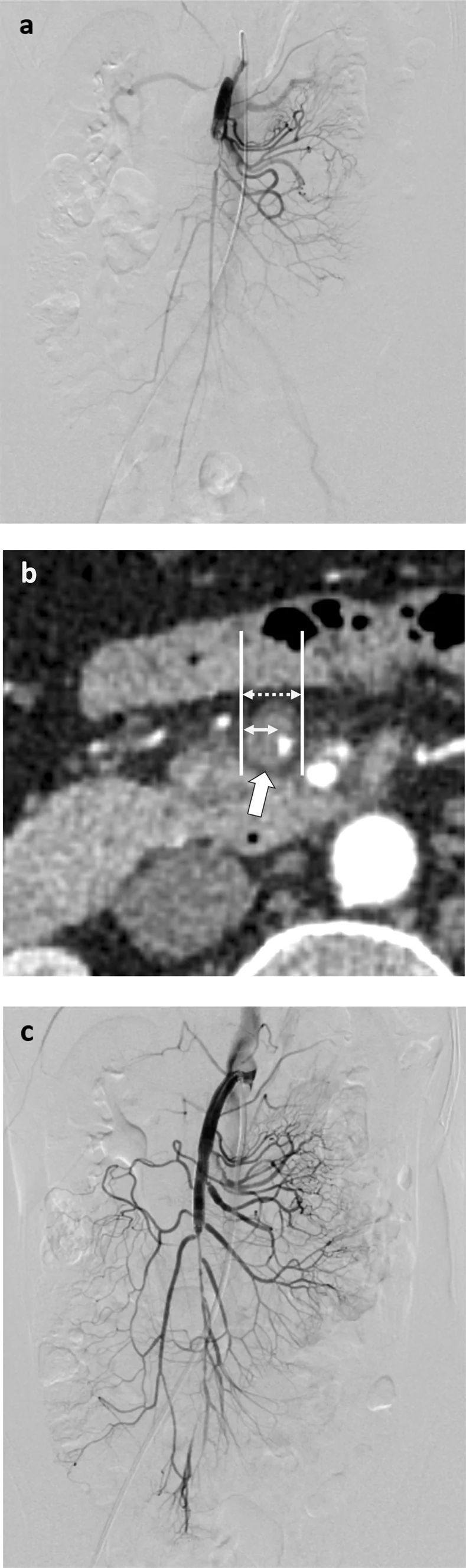
A 47-year-old female patient with ISMAD (Yun’s classification type IIb). **a** Pre-treatment SMA angiography shows poor distal blood flow caused by the dissection. **b** Preoperative contrast-enhanced computed tomography displays SMA with true lumen narrowing caused by the dissection (white arrow). The true lumen stenosis rate was calculated as (1 −  true lumen diameter/total SMA diameter) × 100, corresponding to the false lumen diameter (bidirectional white arrow) divided by the total SMA diameter (bidirectional dotted white arrow). **c** Post-stenting SMA angiography demonstrates improved distal blood flow in the SMA. ISMAD, isolated superior mesenteric artery dissection; SMA, superior mesenteric artery

Morphological analysis demonstrated that Yun’s type III dissection, characterized by true lumen obstruction, was more common in the F-G than in the S-G. Previous studies have emphasized the prognostic importance of true lumen patency in patients with ISMAD. Dong et al. proposed a morphological classification system for ISMAD based on the true lumen residual diameter (TLRD), reporting that patients managed conservatively exhibited a significantly larger TLRD (46.6%) than did those requiring EVT (18.3%) or surgical treatment (0%) [15]. Similarly, Peng et al. demonstrated that patients requiring invasive treatment had a lower TLRD (24.7%) than did those managed conservatively (35.1%) [16]. Therefore, reduced true lumen patency, corresponding to a higher stenosis rate, is likely related to an increased risk of failure of conservative management and the need for invasive intervention (Fig. 3). The exploratory cutoff value of 66.8% stenosis identified in this study should be cautiously interpreted, although it may serve as a clinically intuitive reference point for risk stratification. Because of the limited number of outcome events, the ROC-derived cutoff value may be unstable and should be interpreted as a hypothesis-generating value, not as a definitive clinical threshold. Branch vessel involvement was another significant factor associated with failure of conservative management, consistent with the findings of Hau et al. [17], who identified branch extension as a risk factor for persistent symptoms, and the European Society of Vascular Surgery guidelines [7], which list persistent symptoms as a risk factor for clinical deterioration and failure of conservative management.

**Fig. 4:**
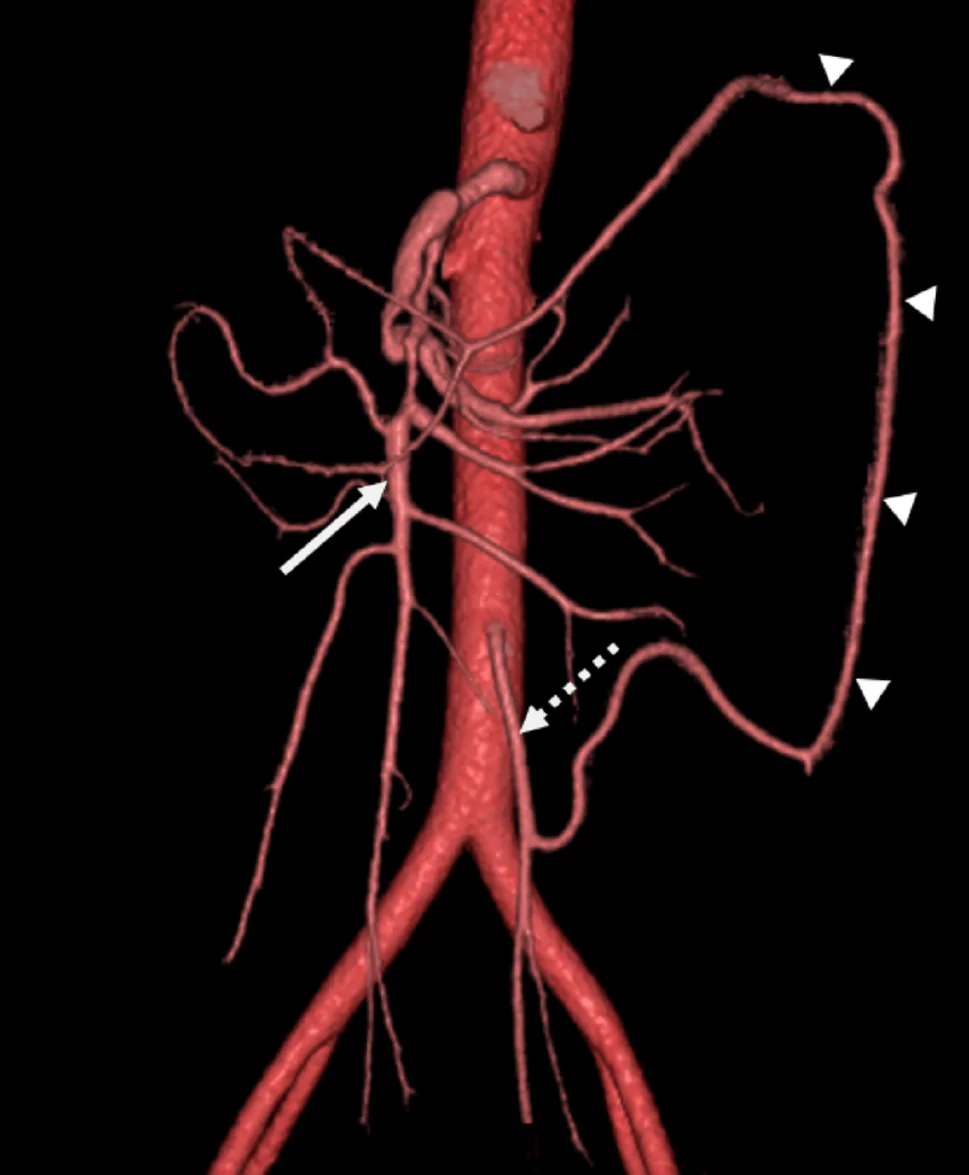
Representative case of RA on arterial-phase contrast-enhanced CT. Three-dimensional volume-rendered image clearly demonstrates a continuous arterial connection between the SMA (white arrow) and IMA (dotted white arrow), consistent with a developed RA (white arrowhead) as defined in this study. RA, Riolan’s arch; CT, Computed tomography; SMA, Superior mesenteric artery; IMA, Inferior mesenteric artery

The role of the RA as a major SMA–inferior mesenteric artery collateral is noteworthy (Fig. 4). RA development likely represents a compensatory response to reduced SMA flow. The RA was visualized in all patients in the F-G in our study, and Peng et al. [16] reported that an RA diameter of > 2.635 mm was associated with the need for aggressive intervention, thereby supporting the notion that a well-developed collateral circulation reflects chronic adaptation to hemodynamic compromise. Because ISMAD often follows a subacute or slowly progressive course, collateral vessels, such as the RA, celiac, and Bühler arteries, may have sufficient time to develop, potentially helping prevent progression to irreversible intestinal changes.

This study has several limitations. First, this was a retrospective study conducted at a single institution with a relatively small sample size, potentially limiting the generalizability of our findings, and multivariable analysis was not feasible. Second, the ROC-derived cutoff should be considered exploratory. In this study, true lumen stenosis was assessed using diameter-based measurements, which are commonly applied in prior ISMAD studies and allow straightforward comparison across patients. Although area-based measurements may theoretically provide a more accurate assessment in cases of eccentric compression, diameter-based evaluation was chosen for its simplicity and reproducibility in routine clinical practice. We acknowledge that this approach may not fully capture complex luminal geometry, and this limitation should be considered when interpreting the results. Additionally, CT-based assessment of RA is inherently dependent on image quality and reconstruction parameters. Therefore, comprehensive clinical judgment remains necessary, and further investigations with a larger cohort are warranted.

Moreover, we focused on the RA among the collateral pathways and did not systematically assess other pathways, such as the celiac or Bühler arteries.

In conclusion, this study demonstrated that both a high true lumen stenosis rate and RA development on CECT are associated with failure of conservative management in patients with ISMAD. The findings suggest that these factors are associated with the clinical course rather than serving as direct predictors of intestinal ischemia, and that their incorporation into clinical assessments may assist in identifying patients at risk of a clinical course requiring closer monitoring or consideration of invasive management.
